# Consumer Behaviour towards Pork Meat Products: A Literature Review and Data Analysis

**DOI:** 10.3390/foods11030307

**Published:** 2022-01-24

**Authors:** Juan Antonio Mondéjar-Jiménez, Francisco Sánchez-Cubo, José Mondéjar-Jiménez

**Affiliations:** Faculty of Social Sciences of Cuenca, University of Castilla-La Mancha. Avenida de los Alfares, 44, 16071 Cuenca, Spain; juanantonio.mondejar@uclm.es (J.A.M.-J.); jose.mondejar@uclm.es (J.M.-J.)

**Keywords:** consumer behaviour, meat, meat product, pork, young consumer

## Abstract

Knowing the behaviour of consumers is essential for all types of companies, including meat companies. For this purpose, academia is an ally of industry, and analysing scientific production seems crucial for conducting future research. Therefore, this study aimed to carry out an exhaustive review of the literature, relying on both descriptive and bibliometric analyses, the latter being through the application of clustering techniques by simple centres. The main results and conclusions are as follows: (1) consumer perceptions, behaviours and attitudes towards food are the main focus of research in this area; (2) the ingredients and additives of meat products are the main concerns in the industry regarding such products; (3) sausages are the dominant meat product; (4) and pork, as well as other types of meat, fall under the generic umbrella term meat. Furthermore, there is a lack of studies considering age, sex and income cohorts. Such lack might have led to finding consumer behaviour and the welfare of animals not significant despite the presupposed positive correlation. The main limitations for researchers are around the availability of budgets and the existence of trade secrets.

## 1. Introduction

Understanding the behaviour of consumers towards a particular product is essential in the marketing strategy of any company worldwide. Of course, this also applies to those businesses devoted to the manufacture and marketing of pork meat products, which are the central axis of this study. Therefore, to achieve their business goals, it is crucial for a company to carry out a detailed analysis of the profiles behaviours of their customers. However, initially, an in-depth study of previous academic works is necessary to gather the extant information regarding meat industry concerns together with consumer behaviours in this field.

In particular, this study aims to focus on the pork meat industry, but a broader scope, including other meat products, is needed nonetheless. That is because a simple search in the *Web of Science* database shows an evident lack in the study of consumer behaviour and pork products. Indeed, the joint search for the terms “pork*”—in the title—and “consumer* behavio*r*”—in the general search—returns only 113 unique articles, but no results are found if narrowing the search by title. Moreover, among these latter results, only 38 papers have been published in the last five years—the period 2017–2021 (accessed on 23 November 2021). Hereafter, the retrieved documents in the aforementioned search are analysed to provide a first overview on the topic, limited exclusively to pork meat and consumer behaviour.

Among these general results, a large part of the records correspond to purely technological aspects—the composition [1,2,3], modification, improvement [4] or health analysis of meat products [5,6]. The latter is a priority issue in developing countries, e.g., South Africa, [7], Vietnam [8,9] or China [10], in which the tradition of selling at street markets makes it difficult to accomplish sanitary regulations.

Similarly, the second largest group of scientific papers deals with consumer perceptions and purchasing behaviours. This has been approached from multiple perspectives, albeit they can be grouped into several subsets of interest. On the one hand, there are numerous recent studies related to the labelling of products and animal welfare—in terms of the adequate expression of information. For the first approach, regarding the labelling in general, the studied points revolve around the traceability of the product, the labelling of its origin and its certification [11,12,13,14,15], including the willingness to pay according to the information and consequences of these certifications [16]. In these studies, the main conclusion is that these factors positively influence the acceptance of the product, but that they are sensitive to other factors, such as price. However, information about health standards shows a residual interest in the literature, since only one of these papers addresses this issue [7]. That might be due to the fact that the bulk of these studies are carried out in developed countries, where consumers assume that the product complies with the guarantees of minimum hygiene–sanitary conditions, and consequently the availability of this information is not relevant for consumers and does not affect purchase intention. For the second approach, regarding the welfare of pigs, the main interest lies in labelling and its acceptance by consumers, which is positive but limited to the information given [17,18]. Moreover, such positive effects only take place if the price difference between products is narrow [19]. Lastly, part of the interest lies in the improvement of the image of the company [20]. Nevertheless, the influence of adequate animal care on the quality of the final product is under-studied, and the findings concerning this relationship were found to be weak [21].

At this point, it must be highlighted that, although developed countries do not face relevant sanitary issues, other problems have arisen. However, the search did not retrieve papers addressing many of them for the specific case of meat and/or pork products. In particular, one of these problems which are not specifically addressed is food waste. This is an issue of great interest due to its importance in the context of the circular economy. In this sense, this topic can be addressed from the perspective of the enterprise [22] or the consumer [23]. Nevertheless, since no results have been retrieved for the pork industry, it seems clear that there is still an evident lack of research regarding pork meat products in the supply chain and households. Therefore, further research is needed to fill this gap, but it is not the aim of this study.

Furthermore, there is an amalgam of academic works that analyse the factors that influence the sale and/or satisfaction with a pork product in its different formats: bacon [24,25], chops [26,27], loin [28], hindquarters [15] or, simply, the acquisition of pork [29,30,31] or Iberian pork [32]. Similarly, the study of consumer perceptions about the characteristics of the product—e.g., flavour, appearance… [33] and about additives is also a relevant aspect in the extant literature. Acceptance of such additives shows a wide range of levels, depending on the case [34,35,36]. Indeed, this issue has even been addressed for artificial meat produced from plants, but without promising results among non-vegetarian or vegan clients [37].

From the above review of the literature about pork meat together with consumer behaviour issues, several lines of research have been identified, which reinforces the idea that knowing the current state of art is essential to continue progressing in market research on pork meat products; for example, past results that have supported contemporary research need to be taken into account for future research. Moreover, this need is reinforced by the scarcity of scientific papers published in the last five years—only 38—but also by the low volume of scientific production since 1972—only 113 documents being available on the *Web of Science*. That shows not only that there are gaps to address as promising lines of research but also that there are gaps that have barely—or never—been tackled. Indeed, this situation entails the aim of study of this piece of work: to compile and analyse most of the extant information regarding the pork industry and consumer behaviour, offering a comprehensive paper which draws an overview of the topic and the main lines for future research.

Considering the above, as well as the scarcity of exclusively pork-related papers, it is necessary to expand the scope of study to other type of meats to cover a larger field that might provide a broader and inclusive view of other factors that have not been investigated in the previously specified search, since it only covered pork-related scientific articles. The aim is to unveil some relationships with the study topic which might be not as evident, to help in defining more promising lines of research. This is why this work is especially relevant in the current scientific context, as it provides a comprehensive, up-to-date, systematic and illustrative review of the scientific literature through a bibliometric analysis.

Thus, after this brief introduction and contextualization of the scientific production generated in the field of the perceptions of consumers of pork meat products and the different factors that this industry encompasses, including technological aspects, an explanation of the materials and methods used in this work is carried out. Then, the obtained results are presented and discussed. Finally, the main conclusions and limitations of this study are extracted.

## 2. Materials and Methods

First, the database used to carry out the bibliometric study was generated by the authors of this work by extracting and filtering the records contained in the *Web of Science* database. To this aim, a sufficiently broad but inclusive search equation was designed to represent the academic literature analysed in the first section of this article. The choice of *Web of Science* as the only database considered for the analysis is due to: (1) it being a leading reputable, worldwide-recognised scientific database, (2) it being the reference scientific index in many countries, (3) it being the most commonly used database for per-forming bibliometric analyses and (4) it not being possible to combine bibliographic data-bases to run the bibliometric software using any of the freely available software. Choosing only one database might lead to some bias since papers not indexed in Web of Science—which mainly affects non-English articles and other databases without impact factor—are excluded. However, this slight bias is commonly assumed and should not significantly affect the results of this study.

The following criteria were taken into account for the design of the study. First, despite including the word *pork* as a term identical to the objective of the work, the authors decided to add the words *meat*, *meat product* and *meat-based product* due to the volume of papers that make comparisons between types of meat or refer to meat products under the same generic umbrella term *meat*. These terms can appear simultaneously, which is often the case for the words *meat* and *meat-based product*. Before this, the term that included the marketing load in the search equation was *consumer behaviour*. In this case, the word *behaviour* was truncated to comprise the English derivation. The rest of the truncations in the equation are due to the inclusion of plural potentials. Finally, a particular interest for companies today was optionally added: attracting young customers [38]. The search returned a total of 3,187 unique articles housed in the *Web of Science Core Collection* (accessed on 23 November 2021). These data are public on this website, and are subject to modifications carried out by the website, either by including new records or by modifying and/or eliminating existing ones. The documents retrieved from the following search equation were converted to an Excel file—which served as input data for the descriptive analysis—and a text file—which served as input data for the bibliometric analysis. This process is automatically performed by *Web of Science*.

“Consumer* behavio*r*” AND (“Meat” OR “Meat product*” OR “meat-based product*” OR “Pork”) OR “young consumer*”

Once the data were obtained, two types of analysis were carried out. First, a descriptive analysis of the extracted documents was performed. This analysis was performed using Microsoft Excel and included the temporal evolution of the articles published from the first record registered in the *Web of Science* (1963) to the *Early Access* articles published in 2022. In addition, the top 15 journals indexed in the *Journal Citation Reports 2020* were analysed through the number of papers published since 1963. This analysis was complemented by the details of the last five years in these journals.

Next, the second analysis consisted of a bibliometric study of the same data retrieved for the descriptive analysis. To this end, the *SciMAT* software [39] was used. This software allows a wide range of analyses in addition to the classic clustering tool, highlighting the pre-processing options. By using this tool, the following inquiries were executed for the purposes of this work. First, a strategic diagram was extracted, which allowed the state of the main topics in the literature to be determined according to their centrality—that is, the strength with which they are related to the rest of the thematic clusters [40]—and their density—that is, the development, according to the chosen unit of measure, of the thematic cluster [41]. Then, the networks of the two main clusters obtained in the strategic diagram were analysed.

In order to facilitate the replicability of the study, the nine steps followed, in the order of the *SciMAT* software User Guide [42] are as follows: (1) the period 1963-2022, which covers all the data extracted; (2) *Words*—*Author’s, Source’s* and *Extracted words* as a unit of analysis; (3) frequency threshold set to two; (4) co-occurrence analysis; (5) network reduction threshold set to two; (6) normalization of the network by similarity by the *Equivalence Index*; (7) selection of the *Simple Centers Algorithm* as the algorithm for the creation of the clusters [43]; (8) choice of the *core mapper* as a visualization tool [44]; and (9) the choice of the *h-index* and *sum citations* as quality measures. The tenth step included in the guide, in relation to the choice of similarity measures for the evolution and *overlapping* maps, was not included since, as there was only one period in this study, they lacked content.

## 3. Results

**Figure 1:**
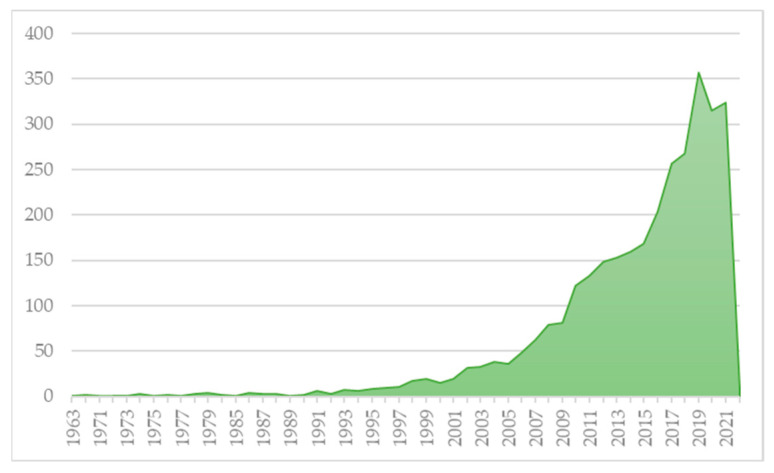
Evolution in the number of papers (1963–2022).

As introduced in the previous section, the first step is the descriptive analysis of the records obtained from the *Web of Science*, which was undertaken using various quantitative approaches. The first is the temporal distribution of the volume of pieces of work in the period 1963–2022, as represented in Figure 1. This trend is similar to the general one, regardless of the subject of study, that is, a significantly low volume of documents until the 1950s and exponential growth from the 1980s [45]. In the case of this work, the graph is very similar but with a considerable time lag, in addition to being, in general, flatter and having more moderate exponential growth. It is worth highlighting the years 2009 to 2010 and 2015 to 2016, where two turning points were identified in the rise of the volume of scientific papers. For the first case, it seems to match the release of two Special Issues in the *Meat Science* journal. For the latter, there is not an evident breaking point for such an increase; it might be due to an organic growth of interest in the topic. The historical maximum of 357 documents in 2019 should also be outlined.

**Figure 2:**
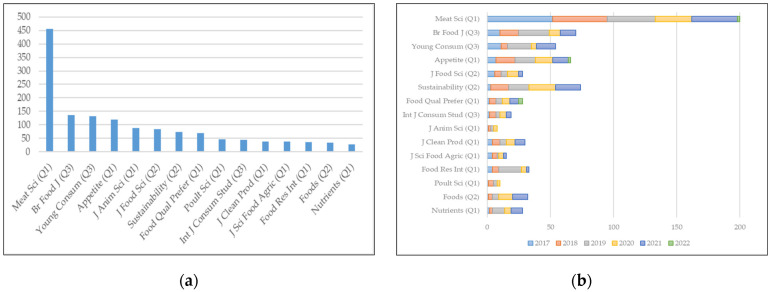
Figures of the main journals in the topic: (**a**) journals with most published papers (1963–2022); (**b**) number of papers published by these journals in the recent years (2017–2022). Both figures’ numbers refer to the papers included in the search equation proposed in Section 2.

In Figure 1, the evolution of the number of articles can easily be seen. Nevertheless, the data retrieved from the scientific database can be further utilised to obtain more detailed and relevant descriptions. In this sense, Figure 2 comprises two panels, which represent the number of articles published by the 15 journals with the highest number of articles published on the subject of study (a) and their distribution in the last five years—2017–2021—and in 2022 to include *Early Access* pieces of work (b). Similarly, the quartile of each journal is indicated in the *Journal Citation Reports 2020*, with the majority of the journals in the first quartile.

Nonetheless, the journal *Meat Science* stands out, with a greater difference compared to the rest, which, with 456 articles in the entire period, accounts for 14.31% of the related scientific production. In addition, as shown in panel (b), in the last five years, it has published 200 articles, that is, 6.28% of the articles in the entire period and 28.78% of the articles published in the last five years. They are followed—quite far behind, but above 100 records—by the *British Food Journal*—4.30%; Young Consumers—4.11%; and Appetite—3.48%.

In addition, panel (b) shows two other notable facts. On the one hand, it is noticeable that the journals *Sustainability, Journal of Food Science, Appetite, Young Consumers, British Food Journal* and *Meat Science* constitute the bulk of the most recent articles and, in a more or less homogeneous way, in terms of the volume of articles published annually. On the contrary, the journal *Food Research International* stands out, with a peak of 18 articles published in 2019, but only five and four in the previous and subsequent years, respectively.

Thus, Figure 1 and Figure 2 show information about the number of articles, their evolution and the journals that published them. The information related to the other two critical aspects in bibliometric analyses, the keywords and the authors, remains unknown. Starting with the latter, the authors were excluded from the study because, given the volume of papers contained in the analysis, the author relationship maps that were generated do not clearly represent some relevant phenomenon or network of authors. Additionally, the analysis of the keywords was omitted in the descriptive analysis, but it was included in the bibliometric analysis described below since it provides more comprehensive information. The data include the keywords given by the authors, those of the journal and the extracted ones.

**Figure 3:**
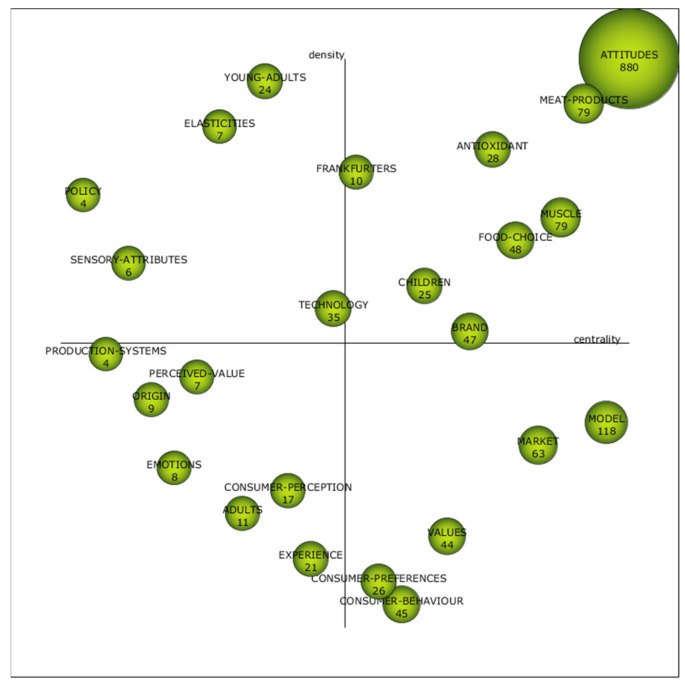
Strategic diagram.

However, the bibliometric analysis began with the generation of the strategic diagram (Figure 3). This diagram was organised around the centrality and density axes, which were previously explained in the *Materials and Methods* section. Following Cobo et al. [39], the topics in the upper right quadrant are the *motor clusters*, that is, the most contemporary and potential topics. On the other hand, the thematic clusters in the lower-left corner are the minority themes, due to the fact that they are either emerging or disappearing. In addition, those in the upper left corner are those that are highly developed but isolated from the rest of the clusters, and finally, those located in the lower right corner are the core and transversal themes. In this regard, Figure 3 presents a relatively homogeneous distribution of the topics. Note that the figure represented in each cluster is the number of scientific papers that it groups together.

Among all the topics, the most prominent is, by far, *attitudes*. This is framed in the close relationship of the terms *attitudes* and *behaviours*, although they are not synonymous. Consequently, this cluster was not omitted. In addition, its proximity to the term *meat products* is positive, as it reinforces the idea that the conjunction of both topics is a current issue and one in which efforts must be invested to contribute to scientific knowledge.

However, the location of the *young adults* cluster is striking since, although it is a high-impact topic, as in food waste issues [46,47], it is isolated from the rest of the clusters, especially *attitudes* and *meat products*. On the other hand, *consumer perception* and *experience* clusters, located in the quadrant of emerging or disappearing issues, were expected to be in the upper right quadrant, as the evolution of the *consumer preferences* and *consumer behaviour* clusters is a core issue in the field of consumer study. Unfortunately, the rest of the clusters identified are spread through the diagram and, in many cases, the documents they include are relatively few. That leads to interesting but self-explained information depending on the quadrant in which the cluster is placed. For the purpose of this study, the most relevant ones have already been commented on, but other authors might find relevant information in other clusters and their position.

**Figure 4:**
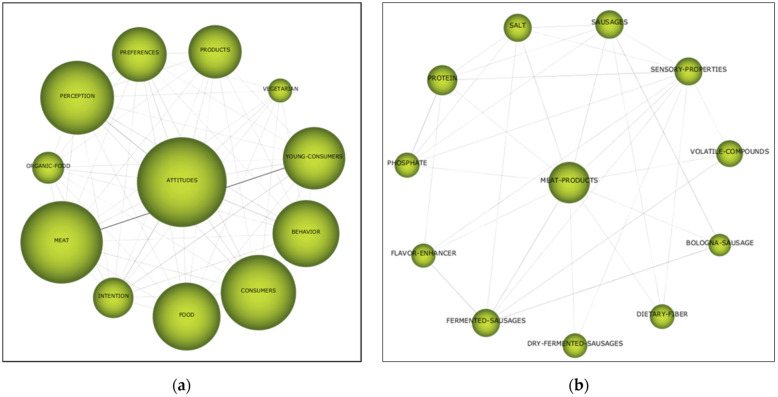
Main clusters: (**a**) “attitudes” cluster; (**b**) “meat products” cluster.

Therefore, Figure 3 shows the importance of *attitudes* and *meat products* clusters as current frontiers of knowledge and hot topics. Consequently, both clusters’ networks were generated (Figure 4) to deepen the study of the connections they represent.

First, the *attitudes* cluster—panel (a)—has two connections that stand out above the rest: the relationships with *meat* and with *young consumers*. In both cases, the intensity of the relationship and the volume of work that they bring together deserve special attention. In fact, this is the reason that they stand out—they are outlined as the most important subjects of all the documents extracted. Similarly, but with less intensity, other relationships are relevant. On the one hand, *perceptions* and *behaviour* concerning *attitudes* are the three possible approaches from which to analyse the *consumer*, precisely, in relation to *food*. This is a clear example of how the interrelationships in the same cluster network explain the phenomenon of interest. That is clearly visible from the size of the clusters and the lines which connect them. Additionally, therefore, it reflects the current state of the study question. However, it might be stated that it represents a vague picture of it, but there the role of scientists in future research might be to delve deeper into each relationship. Indeed, some very important relationships are missed, e.g., consumer behaviour, attitudes or perceptions and their relations to age, gender or income cohorts.

Finally, panel (b) of Figure 4 represents the second most important cluster according to the strategic diagram of Figure 3, that is, *meat products*. In this case, the relationships are homogeneous, and no term stands out above the rest; nevertheless, valuable information was extracted. First, three of the eleven terms refer to *sausages*, which are the only meat product specifically mentioned in the network. Second, the rest of the terms refer to the additives, components or characteristics of meat products, which reflects the concerns of academia and the industry to determine their influence on the products and the consumer’s purchase decision [1,2,3]. Finally, the lack of the term *pork* should be highlighted in both cluster networks—panels (a) and (b)—but especially in that relating to meat products. However, it does not refer to any other type of meat, so it could not be due to the pork itself, but to special interest in the components of the products and the sausages. In consequence, future research should tackle this gap by addressing the issue through other approaches which might exclude additives—e.g., focusing on the quality of additive-free products—and include differentiations between types of meat.

## 4. Discussion

The main objective of this work was to offer a global vision of the academic literature on the behaviour of consumers of meat products. Thus, its purpose is to be a foothold for future research by providing an approximation of the main subjects under study from the outset of related studies. Therefore, the main contribution of this work lies in synthesising the bibliographic information available to offer researchers the academic context in which related research is found.

For this purpose, the authors of this paper used descriptive and bibliometric analysis tools—the latter being especially interesting since it offers more advanced statistical support, through clustering techniques—to the literature review. In this sense, the extensive database extracted, and the detailed overview of the four main variables considered in this type of study—number of articles, reviews, keywords and authors—empirically supports the conclusions that can be drawn from this work.

Thus, it was shown how the development of the literature on the behaviour of consumers of meat products follows a general pattern in academia, albeit with a delay of nearly a quarter of a century—compared to the general trend in the evolution of papers—and with a fairly exponential, but moderate, increase in the last years. In addition, the data show that the journal *Meat Science* is the undisputed leader in the thematic area, having published 6.28% of the articles in the period 1963-2022 and comprising 28.78% of the articles published in recent years—the period 2017–2022. In this sense, it was shown how four other journals are competing regarding the number of publications in recent years. Consequently, these five journals are leading scientific production in the area.

Then, the bibliometric analysis showed two highly illuminating results. First, it places the clusters obtained through a cluster analysis, using the simple centres algorithm, into a strategic diagram that classifies them according to two variables: centrality and density. This allows the identification of which topics are the hottest and which are stagnant or disappearing. It is noteworthy that the clusters *attitudes* and *meat products* are absolute leaders in the quadrant of *motor clusters*. Second, both clusters’ networks are compiled and displayed. As a result, the following can be concluded: (1) consumer perceptions, behaviours and attitudes towards food are the main axes in research in the area; (2) the components and additives of meat products are the main concerns in the industry in relation to such products; (3) sausages are the dominant meat product; (4) and pork, as well as other types of meat, fall under the generic umbrella term *meat*.

Therefore, it can be concluded that, in the sphere in which this work was conducted, more scientific research is needed. Thus, with a focus on pork meat, research would aim to better understand pork consumers, the products they prefer, their characteristics, their willingness to pay, etc. In this regard, and with a view to future research, from the review of the literature carried out, the importance of the context of the studies is drawn, with special attention to the difference between developed and developing countries. There is also a lack of studies devoted to different perceptions according to age, gender and income cohorts. With this in mind, a wide range of future lines of research opens: from analysing differences between consumers depending on their characteristics to studying how differences in meat composition affect purchasing intention.

Nevertheless, some limitations arise in this type of paper. First and foremost, no bibliometric software allows for combining different databases. Therefore, the *Web of Science* database was selected according to its international relevance and its common use for performing bibliometric analyses. Consequently, those papers not indexed in the journal *Citation Reports* or the *Emerging Sources Citation Index* do not appear in the bibliometric analysis or the literature review. Besides, despite the authors’ intention being to design a wide search equation and the best efforts made to provide a comprehensive literature review, some relevant papers may fall out of the search. Lastly, the budgetary and trade secret limitations for developing studies are evident and might hinder future research.

## Data Availability

The data used in this piece of work are publicly available at the *Web of Science* (https://www.webofscience.com, accessed on 23 November 2021) through the aforementioned search. Search results may change due to new papers and/or modifications of the extant ones undertaken by the *Web of Science*.
